# PPARs Signaling and Cancer in the Gastrointestinal System

**DOI:** 10.1155/2012/560846

**Published:** 2012-09-17

**Authors:** Valerio Pazienza, Manlio Vinciguerra, Gianluigi Mazzoccoli

**Affiliations:** ^1^Gastroenterology Unit IRCCS “Casa Sollievo della Sofferenza” Hospital, Viale dei Cappuccini n.1, 71013 San Giovanni Rotondo, Italy; ^2^The Institute of Hepatology 69-75 Chenies Mews London, WC1E 6HX London, UK; ^3^Division of Internal Medecine IRCCS “Casa Sollievo della Sofferenza“ Hospital, Viale dei Cappuccini n.1, 71013 San Giovanni Rotondo, Italy

## Abstract

Nowadays, the study of the peroxisome proliferators activated receptors (PPARs) as potential targets for cancer prevention and therapy has gained a strong interest. From a biological point of view, the overall responsibility of PPARs in cancer development and progression is still controversial since several studies report both antiproliferative and tumor-promoting actions for these signaling molecules in human cancer cells and animal models. In this paper, we discuss PPARs functions in the context of different types of gastrointestinal cancer.

## 1. Introduction 

Since the discovery of the peroxisome proliferators activated receptors (PPARs) [1] in Xenopus frogs as receptors that induce the proliferation of peroxisomes in cells [2], three main forms transcribed from three different genes have been indentified: PPAR*α*, PPAR*β*/*δ*, and PPAR*γ*. Despite the little divergence of homology observed, each isoform possesses distinct biological activities and is expressed in different tissues [3]. PPAR*α* is mainly expressed in the liver, the kidney, and the heart and is primarily involved in lipid metabolism. PPAR*γ* is a master regulator of adipogenesis and fat storage: it regulates adipocyte differentiation and insulin sensitivity in adipose tissue. PPAR*β*/*δ* is found in a broad range of tissues but markedly expressed in brain, adipose tissue, and skin and its function awaits further exploration. PPARs are key mediators of energy homeostasis, lipid, and glucose metabolism although they have also been associated with other biological processes including development, differentiation, inflammation, atherosclerosis, wound healing, and tumor formation. All PPARs heterodimerize with the retinoid X receptor (RXR) to bind successively to specific DNA regions of target genes named PPREs (peroxisome proliferator hormone response elements). Like PPARs, RXR exists as three distinct isoforms: RXR*α*, *β*, and *γ*, all of which are activated by the endogenous agonist 9-*cis* retinoic acid [4]. Contrasting observations confer to PPARs a double-edge sword nature in cancerogenesis, considering that either tumor suppressing or stimulating effects have been evidenced for these nuclear receptors [5].

PPARs function is modified by the specific shape of their ligand-binding domain induced by ligand binding and by a number of coactivator and corepressor proteins, the presence of which can stimulate or inhibit receptor function, respectively [6]. Endogenous ligands for the PPARs include free fatty acids and eicosanoids. PPAR isoform-specific agonists, specifically fibrates for PPAR*α* and thiazolidinediones for PPAR*γ*, are currently prescribed as lipid and glucose-lowering drugs, respectively [7]. Although several reports highlight antiproliferative and prodifferentiative actions of PPAR*γ* ligands in cancer cell lines and animal models of human neoplastic disease [8], more recent studies illustrating tumor-promoting effects of PPAR*γ*, in particular in colon and breast cancer models, raise considerable concern about the practicability and safety of PPAR*γ* ligands as anticancer drugs [9, 10]. In this paper we discuss PPARs functions in the context of different types of gastrointestinal cancer.

## 2. PPARs and Gastrointestinal Tract Cancer 

Numerous studies in the last decade have focused on the effects of PPARs activity on gastrointestinal tract tumor biology, exploring mechanisms, target genes, clinical applications, and evaluating the potential therapeutic use in cancer treatment of PPARs agonists, which seemed promising as components of combination treatments in both *in vitro* and *in vivo *models of cancer [11–13]. In particular, a possible role for PPAR*γ* as a tumor suppressor and as an inducer of differentiation of cancer stem cells has been explored, and its protein level in tumor specimens has been identified as a significant prognostic marker [14].

A recent meta-analysis has found an association between the *PPARG* polymorphism 34 C > G and colon cancer risk [15], and a *PPARG* germline mutation replacing serine 289 with cysteine in the mature protein (S289C) has been reported associated with dyslipidemia and colonic polyp formation progressing to full-blown adenocarcinoma [16]. Furthermore, studies performed in animal models challenged with procarcinogenic and anticarcinogenic agents have put in evidence that PPAR*γ* signaling pathway is critically engaged in the antitumor activity of normal organisms [17]. Anyway, the role of PPAR*γ* in the neoplastic diseases of the gastrointestinal tract remains controversial, as this nuclear receptor shows dissimilar growth-suppressive effects in different cancers. Moreover, PPAR*γ* activation induces diverse growth inhibition in different cancer cell lines [18]. PPAR*γ* inhibits tumor growth only in the presence of functional APC but not in cells with loss of APC function [19], and PPAR*γ* agonists have been reported to have tumor-promoting effects in the Apc^*Min*⁡/+^ mice [10], suggesting that loss of APC may alter the normal response of intestinal epithelial tumor cells to PPAR*γ* agonists. The latter could be one important feature that can explain the discrepancies reported in the literature about the dual role of PPAR*γ* in gastrointestinal cancer.

In the esophagus, the evaluation of PPAR*γ* protein and mRNA expression levels in samples of normal esophageal squamous epithelium, Barrett's esophagus, and esophageal adenocarcinoma has shown a trend toward increased expression going from normal tissue to pathological samples and a trend towards increased PPAR*γ* expression with decreasing levels of differentiation [20]. Similarly, PPAR*γ* expression is increased in human gastric cancer tissue [21], and immunohistochemistry has evidenced its overexpression in gastric mucosal dysplasia and gastric carcinoma compared with chronic gastritis [22]. In addition, the presence of PPAR*γ* protein has been evidenced in surgically resected specimens from well differentiated, moderately differentiated, and poorly differentiated gastric adenocarcinoma [23]. On the other hand, PPAR*γ* agonists show dose-dependent inhibitory effects on the proliferation of gastric cancer cell lines, and this effect is augmented by the simultaneous addition of 9-*cis* retinoic acid; flow cytometry demonstrates G1 cell cycle arrest and a significant increase of annexin V-positive cells, suggesting that induction of apoptosis together with G1 cell cycle arrest may be one of the mechanisms of the antiproliferative effect of PPAR*γ* activation in human gastric cancer cells [23]. 

Regarding the large bowel, high expression of PPAR*γ* is detected in the normal mucosa of the colon and rectum, and a deficiency in intestinal PPAR*γ* is associated with enhanced tumorigenicity in mouse small intestine and colon. A series of evidence suggests that *PPARG* is a tumor suppressor gene in colorectal cancer: (i) loss of function point mutations has been evidenced in one allele of *PPARG* in primary colorectal patients, and the mutations impair the function of PPAR*γ* by affecting the ligand-binding domain, which results in an inability to bind ligands and control gene regulation; (ii) polymorphism in the PPAR*γ* gene has been found in colorectal cancer patients; (iii) expression of PPAR*γ* in colorectal cancer is associated with a good prognosis [24]. Anyway, decreased PPAR*γ* expression compared with adjacent normal colonic mucosa is detected in a number of colorectal cancer patients [25], and *PPARG* inactivation seems to play a role in colorectal cancer progression, although the events involved are not yet clear. In a large series of primary colorectal cancers, about 60% of tumors showed PPAR*γ* upregulation, whereas 35% of the tumours showed lower PPAR*γ* levels compared to the nontumorous normal mucosa. A significant association was evidenced between low PPAR*γ* expression and distant metastases and reduced patients' survival [26].

*PPARG* epigenetic silencing has been found to be coordinated by ubiquitin-like with PHD and RING finger domains 1 (UHRF1), a member of a subfamily of RING-finger-type E3 ubiquitin ligases, which mediates colorectal cancer progression. This protein is encoded by the UHRF1 gene and its expression peaks at late G1 phase and continues during G2 and M phases of the cell cycle, playing a major role in the G1/S transition by regulating topoisomerase II alpha and retinoblastoma gene expression and functioning in the p53-dependent DNA damage checkpoints. UHRF1 binds to specific DNA sequences and recruits a histone deacetylase to regulate gene expression, functioning as a cofactor that coordinates the epigenetic silencing of tumor suppressor genes. UHRF1 overexpression induces *PPARG *silencing through its recruitment on the *PPARG* promoter promoting DNA methylation and histone repressive modifications, and it is associated with a higher proliferative, clonogenic, and migration potential, and with phenotypic features resembling those occurring in the epithelial-mesenchymal transition [27]. PPAR*γ* agonists such as thiazolidinediones, also known as glitazones (rosiglitazone, troglitazone, and pioglitazone), have been shown to induce apoptosis in human colon cancer cells, and the molecular mechanism involves glycogen synthase kinase-3*β* (GSK-3*β*), a crucial activator of nuclear factor-kappa B (NF-kappaB), which plays a critical role in the mediation of survival signals in cancer cells, with inhibition of NF-kappaB activity and GSK-3*β* expression in a dose-dependent manner. Glitazone treatment inhibits colon cancer cell growth, and cells are arrested in G(0)/G(1) phase followed by the induction of apoptosis with concomitant decrease in the expression of the G(0)/G(1) phase regulatory proteins Cdk2, Cdk4, cyclin B1, D1, and E, decrease in the antiapoptotic protein Bcl-2, and increase in the expression of the proapoptotic-associated proteins caspase-3, caspase-9, and Bax [28]. Similarly to the phenomenon evidenced in gastric cancer lines [23], the effect is augmented by the simultaneous addition of the RXR*α* ligand 9-*cis *retinoic acid [29].

On the other hand, inhibiting PPAR*γ* prevents proliferation of human colon cancer HT-29 cells, as evidenced by challenge with cyclic phosphatidic acid (cPA), a structural analog of lysophosphatidic acid (LPA), and a specific, high-affinity PPAR*γ* antagonist [30]. Moreover, synthetic and physiological agonists of PPAR*γ* and PPAR*β*/*δ* induce expression of vascular endothelial growth factor (VEGF) in the colorectal tumor cell lines SW480 and HT29 [31]. Interestingly, PPAR*β*/*δ* is a promising drug target since its agonists promote terminal differentiation, but there are reports showing either pro- or anticarcinogenic effects of PPAR*β*/*δ* in cancer models [32]. Expression of PPAR*β*/*δ* mRNA and protein is lower in human and Apc (+/Min-FCCC) mouse colon tumors in respect of matched normal tissue, and stable overexpression of PPAR*β*/*δ* in human HT29 colon cancer cell lines enhances ligand activation of PPAR*β*/*δ* and inhibition of clonogenicity [33]. The role of PPAR*β*/*δ* in the pathogenesis of colorectal cancer has been evaluated in studies performed *in vivo* on rectal cancer patients and *in vitro* on colon cancer cell lines with different metastatic potentials. The intensity of PPAR*β*/*δ* expression has been found increased in human rectal cancer tissue compared to adjacent or distant normal mucosa [34], in rectal cancers with better differentiation than in those with poor differentiation, and in early-stage tumors than in advanced ones [35]. Besides, PPAR*β* knockdown *in vitro* has evidenced that PPAR*β*/*δ* may facilitate differentiation and inhibit the cell-fibronectin adhesion of colon cancer cell lines [35]. 

Anyway, some colorectal cancer cell lines are resistant to PPAR*γ* agonists, because elevated PPAR*δ* expression and/or activation of PPAR*δ* antagonize the ability of PPAR*γ* to induce colorectal carcinoma cell death, as a result of opposing effects of PPAR*δ* and PPAR*γ* in regulating programmed cell death mediated by survivin and caspase-3: activation of PPAR*γ* results in decreased survivin expression and increased caspase-3 activity, whereas activation of PPAR*δ* counteracts these effects [36]. In addition, the concomitant expression of PPAR *β*/*δ* and cyclooxygenase (COX)-2 in tumor tissues is associated with a higher incidence of liver metastasis and consequent poor prognosis in colorectal cancer patients [37].

PPAR*γ* activation induces expression of Krüppel-like factor (KLF) 4, known also as gut-enriched Krüppel-like factor (GKLF), which acts as a transcriptional activator or repressor depending on the promoter context and/or cooperation with other transcription factors. KLF4 is a nodal player in the network of PPAR*γ*-regulated genes, and treatment of colon cancer cells with PPAR*γ* agonists influences KLF4 target genes, whose expression is decreased (cyclin D1) or increased (GPA33, encoding the glycoprotein A33 that is a colon cancer antigen, p21WAF1/Cip1, and keratin 19), respectively [38].

Epigenetic silencing of *PPARG* in colorectal cancer may be a significant prognostic marker of tumor progression, and methylation on a specific region of the promoter is strongly correlated with PPAR*γ* lack of expression in primary colorectal cancers and with patients' poor prognosis [26]. The same methylation pattern is found in PPAR*γ* negative colorectal cancer cell lines. Transcriptional silencing is due to the recruitment of methyl CpG binding protein 2 (MeCP2), histone deacetylase 1 (HDAC1), and histone-lysine N-methyltransferase (EZH2) that impart repressive chromatin signatures determining an increased cell proliferative and invasive potential [26].

As reported in this section, many clinical and experimental data support the critical role played by PPARs in gastro-intestinal tumorigenesis and neoplastic gut disease behavior, but the molecular mechanisms involved are still a matter of debate. Furthermore, the results of many studies are conflicting and lead to the conclusion that PPARs may have both tumor suppressor and procarcinogenic activity. These controversies may arise from methodological differences among the study protocols, anyway some evidence suggests that ligand-related PPARs activation induces growth arrest in cancer cells and tumor growth inhibition deriving from antiproliferative or proapoptotic effects. On the other hand, PPARs have been found to stimulate tumor cell proliferation and induce neo-angiogenesis, favoring cancer growth and spreading. PPARs agonists provoke several physiological modifications influencing lipid metabolism, glucose homeostasis, and inflammation signaling cascade, and considering that among the major risk factors for colorectal cancer are comprised obesity, metabolic derangement, and chronic inflammatory bowel disease, PPARs modulation could be a valuable tool in the prevention and treatment of colorectal cancer. A mandatory and preliminary condition is represented by the full understanding of the complex mechanisms involved in the regulation of PPARs transcriptional activity and unveiling of the intricacy of PPAR-dependent and PPAR-independent effects stimulated by the different ligands. The same PPAR is able to modulate different target genes and cooperate with other nuclear receptors and signalling molecules involved in cell proliferation and cell death, increasing the difficulty to dissect the role of the single players that take part in this physiologically basic but really intricate network.

## 3. PPARs and Liver Cancer 

Hepatocellular carcinoma (HCC) is the most common type of liver cancer. HCC often arises from viral hepatitis infection (hepatitis B or C), cirrhosis, alcohol consumption being its most common cause. HCC has recently been linked to nonalcoholic fatty liver disease (NAFLD), the hepatic manifestation of obesity and metabolic syndrome. HCC presents with an aberrant lipid metabolism as revealed by quantitative profiling in patient plasma by using ultraperformance liquid chromatography coupled to mass spectrometry approaches [46]. Compared to other cancers, HCC is quite a rare tumor and, in countries where hepatitis is not endemic, most malignant cancers in the liver are not primary HCC but metastasis (spread) of cancer from elsewhere in the body, for example, colorectal cancer. A great bulk of evidence suggests a role for lipid-sensing nuclear receptors in the pathogenesis of NAFLD and HCC. Lipid sensing nuclear receptors, including PPARs, are the master transcriptional regulators of lipid and carbohydrate metabolism and inflammatory responses, thus standing as suitable therapeutic targets for both NAFLD and HCC [47, 48]. In the leptin-deficient ob/ob mouse model of metabolic syndrome, PPAR*γ* is critical for the development of hepatic steatosis, through modulation of its target protein fat-specific protein 27 (Fsp27) [49]. Hepatic transcriptional effects of PPAR*α*, PPAR*γ*, and PPAR*δ* are multiple and recent hypothesis-driven and unbiased genomewide high-throughput approaches in hepatocytes are continuously uncovering new target genes involved in lipid metabolism or confirming established ones, as *ACSL3, ACOX1, SULT2A1, ACADL, CD36, IGFBP1,* and *G0S2* [50]. PPARs, as other nuclear receptors, can be activated in the liver by several hundreds of environmental chemicals and contaminants, and this has been demonstrated to contribute to the process of hepatocarcinogenesis as observed in *in vitro* and *in vivo* rodent models by large screening studies: however, the biological differences between rodents and humans and the distinct mode of actions make it difficult to extrapolate useful information for the clinics and to determine human carcinogenic risk upon exposure to environmental chemicals [51]. PPAR*α* plays a dominant role in hepatocarcinogenesis induced by trichloroethylene (TCE), an industrial solvent and a widespread environmental contaminant [52]. Beinga central regulator of triglyceride homeostasis and mediating hepatocarcinogenesis in rodents, not surprisingly PPAR*α*, contributes to steatosis and HCC induced by hepatitis C virus (HCV) in rodent models [53, 54]. In human hepatocarcinoma cells, PPAR*α* is chiefly related to apoptosis as evidenced by determination of BAD, myc, and protein phosphatase 2A protein content and PPAR*γ* is instead chiefly related to cell proliferation, evidenced by decreased cell number and increased number of cells in the G0/G1 phase of the cycle [55]. Mice lacking one allele of *PPARG* were more susceptible to liver cancer in a diethylnitrosamine (DEN)-induced HCC model: PPAR*γ* suppressed tumor cell growth through reducing cell proliferation and inducing G(2)/M phase arrest, apoptosis, and upregulating growth differentiation factor-15 [56]. Consistently, troglitazone, a PPAR*γ* ligand, inhibited growth and induced apoptosis of HepG2 cells in a dose-dependent manner [57]. Moreover, in the partial hepatectomy rat model of liver regeneration, it was shown that PPAR*γ* signaling is a key negative regulator of hepatocyte proliferation and may be responsible for the inhibition of liver growth during regeneration [58]. PPARs actively crosstalk with other signaling mediators implicated in lipid metabolism and hepatocyte malignancy; for instance, AMP-activated protein kinase (AMPK), an energy sensing enzyme implicated in the transition from NAFLD to HCC [59], and whose activation has been reported to be lipid lowering and antitumoral in mice and in hepatoma cells [60, 61]. In HCC cells, AMPK activators AICAR and metformin inhibit directly transcriptional activities of PPAR*α* and PPAR*γ* to modulate energy generation through fatty acid oxidation process [62]. Mice with a combination of genetic inactivations for hepatic growth hormone and glucocorticoid receptor signaling effectors displayed upregulation of prolipogenic PPAR*γ* and downstream transcription factor SREBP-1c, demonstrating a crosstalk between these molecular networks [63]. Mice with specific inactivation of the NF-kappaB essential modulator gene (NEMO (L-KO) mice) exposed to a high-fat diet display a worsened liver steatosis as a consequence of PPAR*α* and increased PPAR*γ* expression [64]. From a therapeutic perspective, PPAR*γ* agonists, such as antidiabetic thiazolidinediones (TZD), have *in vitro* antiproliferative effect, have been associated with lower risk and a better prognosis in HCC, not only related to anti-NAFLD but also to antiviral hepatitis effects [65]. The effective anticancer properties and the underlying molecular mechanisms of these drugs *in vivo* remain unclear because the primary target of TZD is PPAR*γ*, which is upregulated in HCC and seems to provide tumor-promoting responses. Reconciling this discrepancy, it may be that these established PPARs agonists exert a hypolipidemic and antitumoral action in liver cells through PPAR-independent pathways [66, 67]. 

 As mentioned, when the liver is infected with hepatic viruses, this can ultimately result in liver cancer, and hepatitis viruses are one of the leading causes of chronic liver disease [68]. Hepatitis viruses are a global health problem if we consider approximately 200 million patients carrying a chronic HCV infection and about 350 million chronically infected with HBV [69].

PPARs were suggested as new therapeutic targets in the traditional treatment of HCV-induced liver injury when two studies found that PPAR*α* drastically decreased in HCV-infected patients [70] together with its target gene carnitine palmitoyl acyl-CoA transferase 1A (CPT1A) [71]. The impaired PPAR*α* expression was due to HCV core protein expression [71]. Successively, we and others have recently uncovered a role for PPAR*γ* in HCV infection [42, 72, 73]. Granted that HCV is classified in six different major genotypes and that mechanisms involved in pathobiology of disease are genotype dependent [39, 73], from a biological point of view, reduced PPAR*γ* levels found in *in vitro* models of HCV expressing the core protein genotype 3a are associated with increased fat accumulation and impaired insulin signaling [72, 73]. The latter impairs the sustained response rate to peg-interferon plus ribavirin in chronic hepatitis C patients [40]. PPAR*γ* degrades IRS1 protein through suppressor of cytokine signaling protein 7 (SOCS-7) whose expression could be pharmacologically controlled by agonist and antagonist of PPAR*γ* [41]. PPAR*γ* agonists have already been suggested as an adjuvant therapy in chronic hepatitis C [74, 75]. In fact, there is the belief that correcting insulin resistance is a rational option in chronic hepatitis C patients [76]. However, new modalities of this correction have to be explored based on the mechanisms inducing insulin resistance, as insulin-sensitizing therapy should be tailored according to the infecting HCV genotype, as suggested [76].

Steatosis is a common histological feature of chronic infection between hepatitis C and B virus. Another common feature is the ability of both viruses in modulating PPAR*α* and PPAR*γ* activity/expression which are related to steatosis. As for HBV, *in vitro* studies using hepatoma cell lines and studies on transgenic mouse models for HBV have provided indication for a role of PPARs in HBV-related diseases and in controlling viral transcription and replication. Kim et al. [77] demonstrated that SREBP-1 and PPAR*γ* were transcriptionally induced by HBV X protein (HBx) in order to provoke hepatic steatosis in HepG2-HBx stable cells and HBx-transgenic mice.

Moreover thiazolidinediones (TZD, class of PPAR*γ* ligands) have been suggested as useful drugs for HCC chemoprevention and treatment as TZD administration in hepatitis B virus (HBV)-transgenic mice reduced tumor incidence in the liver, inhibiting hepatocyte proliferation and increasing apoptosis, probably through inhibition of nucleophosmin (NPM) protein and mRNA expression [68]. Furthermore it was also reported a role for PPARs in regulating HBV transcription and regulation *in vivo* [78] and *in vitro* [79]. Guidotti et al. [78] demonstrated that HBV transgenic mice treated with two synthetic PPAR*α* ligands (Wy-14,643 and clofibric acid) resulted in an increased HBV transcription rates suggesting that in patients receiving these drugs who are also infected with HBV viral replication may be activated, and this could have potentially detrimental effects on the outcome of the viral infection. Conversely, Wakui et al. [79] demonstrated that the PPAR*α* ligand bezafibrate had no effect on HBV replication within HepG2 cells whilst a PPAR*γ* ligand, rosiglitazone, reduced the amount of HBV DNA, hepatitis B *surface* antigen (HBsAg), and hepatitis B *e* antigen (HBeAg) in the culture supernatant, suggesting that the combination therapy of rosiglitazone and nucleot(s)ide analogues or interferon could be a therapeutic rational option also for chronic HBV infection. 

## 4. PPARs and Pancreatic Cancer 

Pancreatic cancer (PC) is one of the most lethal malignant diseases with a really terrible prognosis and is ranked as the fourth leading cause of cancer-related deaths worldwide [80]. PC is referred to as a “silent killer” because early pancreatic cancer often does not cause symptoms and the later symptoms are usually nonspecific and varied. Despite many advances in modern medicine, the available therapeutic strategies based on surgery and conventional chemotherapy are still largely unsatisfactory in patients with pancreatic cancer. When patients present locally advanced or metastatic tumors (which render them ineligible for surgical resection), they are treated with the gold standard chemotherapy which is based on gemcitabine, an S-phase nucleoside cytidine analogue. The overall survival is unacceptably small, and novel therapeutic approaches to overcome the resistance of PC to conventional anticancer therapies are urgently needed. Scientists are also looking for an ideal combination partner in therapeutic settings that require the inhibition of tumor-protecting mechanisms/proteins to overcome treatment resistance. 

PPAR*γ* is commonly upregulated in pancreatic ductal adenocarcinoma and might be considered a prognostic marker in this disease [81]. 

To date several research groups have demonstrated the ability of thiazolidinedione (TZD, class of PPAR*γ* ligands) to attenuate the growth of pancreatic cancer cells *in vitro*, which was associated to G1 cell cycle arrest and cell differentiation and to increased apoptotic cell death [43]. Moreover, Hashimoto et al. [82] suggest a double beneficial effect of TZD showing the dual advantage of inhibiting pancreatic cancer cell growth while reducing the invasiveness of the tumor cells. Moreover, TZD attenuated pancreatic cancer cell migration and invasion by modulation of actin organization and expression of matrix metalloproteinase-2 and plasminogen activator inhibitor-1, respectively [83, 84]. An increasing number of studies have implicated STAT activation, particularly STAT3, in transformation and tumor progression. Direct targeting of STAT3 in malignant tumors may represent another important therapeutic tool as STAT proteins are emerging as ideal targets for cancer therapy [44]. Vitale et al. [85] showed that, in pancreatic cancer cells, PPAR-*γ* agonist (troglitazone, TGZ) counteracts STAT3 protein potentiating the anticancer effects of IFN-*β* through the induction of cell cycle perturbations and the occurrence of autophagy cell death in pancreatic cancer cells. Co-incubation of pancreatic cancer cells with IFN-*β* and TGZ suppresses STAT3 activation and delays G0/G1-S phase progression that occurred together with an increase in p21 and p27 protein expression that was more evident after 24 hours of treatment with the pharmacological combination.

**Figure 1:**
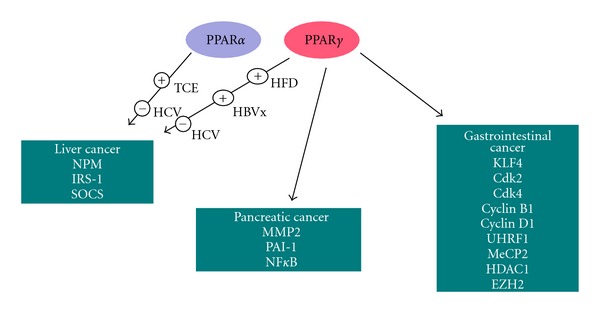
Schematic representation of the PPARs signaling operating in cancer. Krueppel-like factor 4 (KLF4); cyclin-dependent kinase (2, 4); cyclin B1, D1; ubiquitin-like, containing PHD and RING finger domains, 1; methyl CpG binding protein 2 (MeCP2); Histone deacetylase 1(HDAC1); histone-lysine N-methyltransferase (EZH2); matrix metallopeptidase 2 (MMP2); plasminogen activator inhibitor 1 (PAI-1) nuclear factor-kappaB (NF*κ*B); nucleophosmin (NPM); insulin receptor substrate 1 (IRS-1); suppressor of cytokine signal (SOCS). For further explanations, please refer to the text.

Even though we did not observe a PPAR*γ* altered expression in 30 matched pairs of tumour and adjacent normal tissue samples collected from patients undergoing pancreatic resection [45], a recent study supports a role of PPAR*γ* as an ideal partner of the standard therapy based on gemcitabine since the anticancer effect of gemcitabine can be enhanced by ligands for PPAR*γ* such as pioglitazone (Pio) and rosiglitazone [86]. The authors demonstrate that Pio significantly inhibits the NF-*κ*B transcriptional activity and potentiates the gemcitabine effect on the apoptosis rate in three different pancreatic cancer cell lines as demonstrated by cotreatment with Pio and Gem on caspase-3 and caspase-7 cleavage. The authors conclude that since Pio is widely used in the treatment of diabetes mellitus, it may become a possible partner of Gem-based chemotherapy. Considered the adverse effects associated with TZDs, such as weight gain, macular edema, bone loss, and heart failure in at-risk individuals [87, 88], scientists must press on investigating new analogs of PPAR*γ* agonists in order to potentiate the beneficial effect while reducing the side effects (Figure 1 and Table 1).

## 5. Conclusion 

A potential role for PPARs agonists in the adjuvant treatment of digestive system cancers is advisable, but further studies are warranted in order to better clarify the role of PPARs in gastrointestinal cancerogenesis. PPARs could have prognostic and/or therapeutic roles, but there is urgent need to shed light on the favorable potential or harmful risk of their modulators.

## Figures and Tables

**Table 1:** Differential patterns of PPARs expression in gastrointestinal system disease.

Organ	PPARs expression		Author and reference
Esophagus	PPAR*γ* ↑	PPAR*γ* overexpression influences the development of Barrett's esophagus and esophageal adenocarcinoma	Wang et al. [20]

Stomach	PPAR*γ* ↑	Crucial role of PPAR*γ* in the pathogenesis of gastric carcinoma	Ma et al. [21]
	PPAR*γ* ↑	PPAR*γ* is upregulated in gastric adenocarcinoma	Yao et al. [22]
	PPAR*γ* ↑	PPAR*γ* protein evidenced in gastric adenocarcinoma specimens and PPAR*γ* agonists show dose-dependent inhibitory effects on the proliferation of gastric cancer cell lines	Sato et al. [23]

	PPAR*γ* ↑	PPAR*γ* expression in colorectal cancer is associated with a good prognosis	Dai and Wang[24]
Colon-rectum	PPAR*γ * **↓**	PPAR*γ* underexpression is detected in a number of colorectal cancer patients, and epigenetic silencing of PPAR*γ* is a biomarker for colorectal cancer progression and adverse patients' outcome	Pancione et al. [26]
	PPAR*γ * **↓**	PPAR*γ* epigenetic silencing is coordinated by UHRF1 mediating colorectal cancer progression, and a significant low PPAR*γ* expression is associated with distant metastases and reduced patients' survival	Sabatino et al. [27]

Liver	PPAR*α* and PPAR*γ*↓	HCV decreases PPARs in order to induce triglycerides accumulation	Ripoli and Pazienza[39]
			Romero-Gómez et al.[40]
			Pazienza et al.[41]
	PPAR*γ* ↑	HBx enhances C/EBP**α**that in turn induces PPAR**γ** expression and activation	Dharancy et al.[42]
			Tsujie et al.[43]

Pancreas	PPAR*γ* ↑	PPAR*γ* is highly expressed in pancreatic cancer and is associated with shorter overall survival times	Yu and Jove[44]
	PPAR*γ* —	PPAR*γ* is unaltered in PC but expression levels between PPAR*γ* and DNMT1 and between DNMT1 and DNMT3B are highly correlated	Pazienza et al. [45]
